# Positive Impact of Thermal Manipulation During Embryogenesis on Foie Gras Production in Mule Ducks

**DOI:** 10.3389/fphys.2019.01495

**Published:** 2019-12-12

**Authors:** William Massimino, Stéphane Davail, Marie-Dominique Bernadet, Tracy Pioche, Annabelle Tavernier, Karine Ricaud, Karine Gontier, Cécile Bonnefont, Hélène Manse, Mireille Morisson, Benoit Fauconneau, Anne Collin, Stéphane Panserat, Marianne Houssier

**Affiliations:** ^1^INRA, E2S UPPA, UMR 1419, Nutrition, Métabolisme, Aquaculture, Université de Pau et des Pays de l’Adour, Pau, France; ^2^UE-PFG-UE89, Unité Expérimentale sur les Palmipèdes à Foie Gras, Centre INRA Bordeaux-Aquitaine, Benquet, France; ^3^GenPhySE, INRA, ENVT, Université de Toulouse, Castanet Tolosan, France; ^4^UMR-BOA, Centre INRA Val de Loire, Nouzilly, France

**Keywords:** embryonic thermal programing, liver steatosis, mule ducks, lipogenesis, liver fattening, foie gras

## Abstract

Animal studies have shown that very early life events may have programing effects on adult metabolism and health. In this study, we aim, for the first, time to elucidate the effects of embryonic thermal manipulation (TM) on the performance of overfed mule ducks, in particular for the production of foie gras (fatty liver). We designed three embryonic TMs with different protocols for increasing the incubation temperature during the second part of embryogenesis, to determine whether hepatic metabolism could be “programed” to improve its fattening response to overfeeding at the age of three months. Initial results confirm that an increase in the incubation temperature leads to faster development (observed for all treated groups compared to the control group), and a decrease in the body surface temperature at birth. Thereafter, in a very innovative way, we showed that the three TM conditions specifically increased liver weights, as well as liver lipid content after overfeeding compared to the non-TM control group. These results demonstrate that embryonic TM effectively “programs” the metabolic response to the challenge of force-feeding, resulting in increased hepatic steatosis. Finally, our goal of improving foie gras production has been achieved with three different embryonic thermal stimuli, demonstrating the high reproducibility of the method. However, this repeatability was also perceptible in the adverse effects observed on two groups treated with exactly the same cumulative temperature rise leading to a reduction in hatchability (75 and 76% vs. 82% in control), in addition to an increase in the melting rate after cooking. These results suggest that embryonic thermal programing could be an innovative and inexpensive technique for improving foie gras production, although the specific protocol (duration, level or period of temperature increase), remains to be elucidated in order to avoid adverse effects.

## Introduction

The use of a specific stimulus during a developmental period with high plasticity, often during embryogenesis, has already been characterized as potentially leading to a major phenotypic change in response to a specific challenge encountered later in life (Lucas, 1998). This process, called “programing,” can have interesting results in terms of animal health, and may even lead to commercial benefits for the agri-food industry, as illustrated below.

In chickens, high mortality has been observed during exposure to heat stress, resulting in lower profitability in the broiler meat production sector. This observation led researchers to test whether embryonic thermal programing could improve the response of animals to a thermal challenge. They demonstrated that an increase in incubation temperature during the maturation of the hypothalamus-hypophysis-thyroid axis (related to thermoregulation) improves the thermotolerance of broilers by reducing mortality by 50% during a subsequent heat challenge at the age of 35 days (Piestun et al., 2008a). This was manifested by a drop in body temperature associated with a decrease in the plasma content of triiodothyronine hormone (T3) from hatching until age of slaughter (Loyau et al., 2013, 2015; Piestun et al., 2013). Interestingly, this embryonic thermal manipulation (TM) also resulted in an increase in muscle weight at 70 days of age, in the absence of any subsequent heat challenge, leading to enhanced meat production whose benefits are estimated at nearly 1 billion dollars (Piestun et al., 2013). These experiments demonstrate the strong impact that embryonic TM can have on systemic metabolism throughout the life of poultry.

Interestingly, the programing process may also use different stimuli between embryogenesis and a subsequent challenge, resulting in a different phenotypic response. For example, it has been shown that a nutritional stimulus during embryogenesis in chicken (by *in-ovo* feeding) may affect the ability to cope with a heat stress at 9 days of age (Han et al., 2019). In ducks (Pekin breed), it has recently been shown that a continuous rise in incubation temperature results in an increase in liver weight at 2 weeks post-hatching (Liu et al., 2015) and an increase in the activity and expression of hepatic fatty acid synthase (FAS), a key lipogenesis enzyme (Wang et al., 2014). These results suggest that embryonic TM in ducks could enhance lipid storage in the liver, and they inspired us to “program” ducks used for foie gras production (mule breed), in order to improve their response to overfeeding at three months of age.

To produce foie gras, male mule ducks (Marie-Etancelin et al., 2015) are subjected to a 9 to 12-day period of overfeeding (OF) in which large amounts of corn are administered twice a day. This high-carbohydrate diet strongly stimulates hepatic *de novo* lipogenesis and induces liver steatosis (Mourot et al., 2000; Chartrin et al., 2006; Saez et al., 2008; Hérault et al., 2009; Tavernier et al., 2017), characterized by a huge increase in lipid content, ranging from 5% in lean animals to 50–60% in overfed animals (Hermier et al., 2003). We consider that if embryonic TM in mule ducks positively “programs” the hepatic metabolism during overfeeding, we may have discovered a new, simple and inexpensive way to increase foie gras productivity and reduce the number of meals ingested by speeding up fattening of the liver.

We finally designed three new embryonic TM protocols for mule ducks, based on the different experiments mentioned above (in chickens and Pekin ducks) while trying to avoid the adverse effects on hatching and survival that are sometimes observed (Piestun et al., 2008a; Al-Zhgoul et al., 2013). In parallel with a control group incubated at 37.6°C, we tested three warming conditions – continuously (+ 1°C 24 h a day) or discontinuously (+ 1°C 16 h a day and + 1.5°C 16 h a day) – during 50% of the total incubation period (between the 13th and 27th embryonic days). The hatching data were first analyzed and then, the response to force-feeding at the age of 3 months in mule ducks was compared between the control and the thermally manipulated ducks. In order to evaluate the impact of the TM on overall plasma content (thyroid hormone and metabolites), blood samples were taken during slaughter.

## Materials and Methods

### Ethics Approval Statement

All experimental procedures conformed to the French national guidelines on the care of animals for research purposes. The protocols were approved by the Committee for the Care and Use of Animals in the Greater South-Western France region (no. 73) and finally authorized by the Ministry under the file reference APAFIS14196-201805250850236-v3. The present study was carried out in the certified Experimental Station for Waterfowl breeding (INRA, Artiguères, France) – accreditation number B40-037-1.

### Animals

**FIGURE 1:**
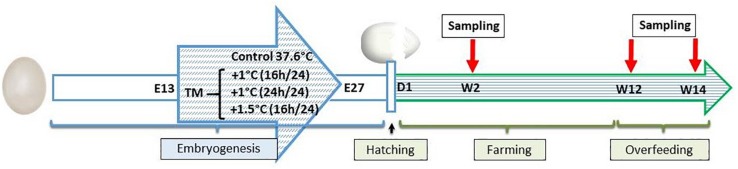
Experimental design. Control eggs were incubated in standard conditions [37.6°C and 47% relative humidity (RH)]. Thermal manipulation (TM) conditions correspond to 38.6°C 16 h/d or 38.6°C 24 h/d or 39.1°C 16 h/d with 63% RH from embryonic day E13–E27.

A total of 2,000 eggs of mule ducks (genotype H85, provided by Grimaud Frères Selection company, Roussay, France) were kept at room temperature for 3 days prior to incubation and randomly assigned to four incubators, thus determining the four treatment groups (500 eggs each, in four incubators). The control group was maintained at 37.6°C and 47% average relative humidity (RH) throughout the entire incubation period. Thermal manipulations (TMs) occurred during the last 14 days of the incubation period, i.e., from the 13th to the 27th embryonic days (E13–E27) at 38.6°C, 16 h/day (+ 1°C 16 h/day), 38.6°C, 24 h/day (+ 1°C 24 h/day), and 39.1°C 16 h/day (+1.5°C 16 h/day) (Figure 1), with RH set at an average of 63% for all manipulated groups in order to avoid egg dehydration. These experimental conditions corresponded to a temperature rise of +224°C for the first treated group (1°C × 16 h × 14 days), and +336°C for the second and third TM groups (1°C × 24 h × 14 days and 1.5°C × 16 h × 14 days) compared to the control group.

All eggs were turned through 90° every 3 h. In each incubator, the temperature and hygrometry were continuously measured by a sensor equipped with remote probes (KIMO, KH200). Unfertile eggs were spotted by candling at E10 and removed from the incubators, with the remaining eggs then being closer together to avoid local temperature disturbance. At E27, all eggs were placed in the same hatcher at 37.3°C and 80% RH. Newly hatched ducks were recorded every day from E27 to E31.

Male ducklings were divided into two groups of 70 for each treatment; they were raised under the same conditions of light and temperature, and fed *ad libitum* from hatching to 4 weeks of age with a starting diet (2,800 Kcal, 17.5% crude protein). From 4 to 8 weeks of age, ducklings were fed *ad libitum* with a growing diet (2,800 Kcal, 15.5% crude protein), and hourly rationed between 8 and 12 weeks of age. At 12 weeks, all ducks were overfed with corn meal twice a day (53% corn and 47% water, Palma Maisadour), for 11 days (21 meals). The body weights and surface temperatures (Evolupharm infrared thermometer “Evoluscan+,” accurate to ±0.1°C), with an average of two consecutive measurements on the rump without feathers), were recorded on days 1, 31, 45, 59, 70, before (D83) and after (D95) overfeeding (OF). The average daily gains were determined at these periods. Ducks were killed by bleeding after stunning in an electric water bath in line with the European Council Regulation (Ec) No 1099/2009 (2009) (Council Regulation (EC) No 1099/2009 of 24 September 2009 on the protection of animals at the time of killingText with EEA relevance) at the Experimental Station for Waterfowl breeding (INRA, Artiguères, France).

The daily feed intake was determined collectively (total feed consumption divided by the number of individuals) until D83 (mean of two collective pens per group) and individually between D84 and D95, corresponding to the OF period.

The feed conversion ratio (FCR) and liver-feed conversion ratio (L-FCR) were determined as the ratio between the individual cumulative consumption and average weight gain and liver weight gain (difference between individual liver weight after OF and mean liver weight per group before OF), respectively.

### Collection of Samples and Plasma Analyses

At days 83 and 95 post-hatching, 40 and 50 males per group, respectively, were randomly selected and slaughtered. Blood samples were collected, from all ducks in disodium EDTA tubes after carotid section. Individual plasma was separated by centrifugation at 2,000 *g* for 10 min at 4°C. Plasma samples were frozen at −20°C for further analysis. Glycemia, triglyceridemia, cholesterol levels and non-esterified fatty acids (NEFA) were quantified by the colorimetric method using an enzymatic kit (Glucose GOD-POD, SOBIODA; Tryglicerides GPO POD, SOBIODA; Cholesterol LD M, SOBIODA, NEFA HR2, Wako, Richmond, United States). Radioimmunoassays of free thyroxin (T4), free triiodothyronine (T3) (RIA FT3 and FT4, Beckman Coulter) were applied to the plasma samples. The intra and inter-assay variations (CV) were 6.4 and 5.5% respectively for T3 and 10.29 and 7.58% respectively for T4. Plasma corticosterone concentrations were measured using a commercially available double antibody RIA-kit (MP Biomedicals, NY, United States) with intra and inter-assay (CV) of 7.1 and 7.2%, respectively.

After dissection, the liver, breast muscle, leg muscles, abdominal fat and subcutaneous adipose tissue (SAT) were weighed. Pieces of liver were sampled in the middle of the large lobe for the study of liver lipid content and were stored at −80°C, or immediately fixed in 4% paraformaldehyde for histological analysis.

### Liver Lipid Content, Dry Matter, and Melting Rate

Near Infra-Red Spectra (NIRS) were collected in absorbance from 350 to 2,500 nm with an interval of 1 nm using the Labspec^®^ 5000 Pro spectrometer (ASD Inc., Boulder (CO), United States) to predict biochemical liver characteristics as described below. A piece of 15 g of each liver was quenched in liquid nitrogen and stored at −80°C. After grinding in liquid nitrogen, samples were desiccated in an oven at 105°C for 24 h (JOCE, 1971) to record the dry matter content. For 40 livers collected before the overfeeding period, the total lipid content was measured by extracting all lipids from a 2.0 g sample by homogenization in chloroform methanol 2:1 (v/v) and measured gravimetrically according to the Folch et al. method (Folch et al., 1957). For 50 fatty livers, the same protocol was applied to a 0.3 g sample. The total lipid content was then predicted for all samples by using a prediction equation developed on NIRS spectra according to the method described by Marie-Etancelin et al. (2014) using previous analyses. The spectrum data were shortened from 650 to 2,350 nm and transformed via a Standard Normal Variate and Detrend 1, 10, 10, 1 normalization. The prediction equation was then based on a modified Partial Least Square (PLS) analysis of the Winisi^®^ software (version 4.6.8, FOSS Analytical A/S, Hilleroed, Denmark).

For the melting test, 60 g samples were taken from the middle part of the livers and placed in a tin can used for the melting test. The tin cans were cooked in an autoclave at 85°C for 60 min. The cans were subsequently chilled using tap water and stored at 4°C for 2 months to mimic the mean commercial storage period. After this storage period, the cans were heated in a water bath (75–80°C, 20 min), the melted fat was removed, and the remaining sample was placed on absorbent paper. The melting rate was then measured as the percentage of loss from the initial liver weight.

### Histological Analysis

Liver samples (seven per group) were fixed with 4% paraformaldehyde, embedded in paraffin and cut into 10 μm thick sections. Microscope slides were stained using Masson’s Trichrome. Liver sections were scanned with a Panoramic scanner 250 from 3dHistech with 40× magnification. The degree of steatosis, hepatocyte ballooning and fibrosis was scored using Masson’s Trichrome-stained liver sections. Each variable was graded from zero (none) to three (severe). ImageJ 1.52 software was used to measure the lipid droplet diameter in each group (mean of 2 representative fields per slide, *n* = 7 liver sections per group). The image was first converted to 16 bits in black and white, then an automatic threshold was applied with a dark background, and the area of circular objects over 48 μm^2^ was measured automatically. A binary watershed was used to separate touching droplets and objects touching the edge of the image were removed.

### Statistical Analysis

Statistical analyses were carried out using the GraphPad Prism version 8 for windows [GraphPad software, La Jolla, CA, United States^1^ (serial number GP8-1598457-RJQD-5E2EC)]. When the dataset had a Normal distribution (assessed by Shapiro–Wilk test), the variances of the four groups were tested with a one-way ANOVA to determine whether the observed factor (TM) had a significant impact on the measured parameter. In the event of a significant difference, a Tukey *post hoc* multiple comparison test was performed to identify the differences among the means of the four groups. When the Normal distribution was not demonstrated, a Kruskal–Wallis test was performed as a non-parametric variance analysis followed by a Dunn test as a *post hoc* multiple comparison analysis.

When two factors were tested on the same graph (OF and TM), the variances were first tested with a two-way ANOVA (with treatment: control vs. TM, and sub-treatment: Pre-OF vs. Post-OF). In the event of a significant difference, a Tukey *post hoc* analysis was then performed to compare all groups with each other. The model includes an interaction test between these effects.

Hatchability and sex ratio data were analyzed by the chi-square test.

The data are presented as the average ± standard error of mean (SEM). In every case, differences between the groups were considered statistically significant if the value of *P* < 0.05.

## Results

### Zootechnical Data From Hatching Up to Day 17 After Hatching (D17)

**FIGURE 2:**
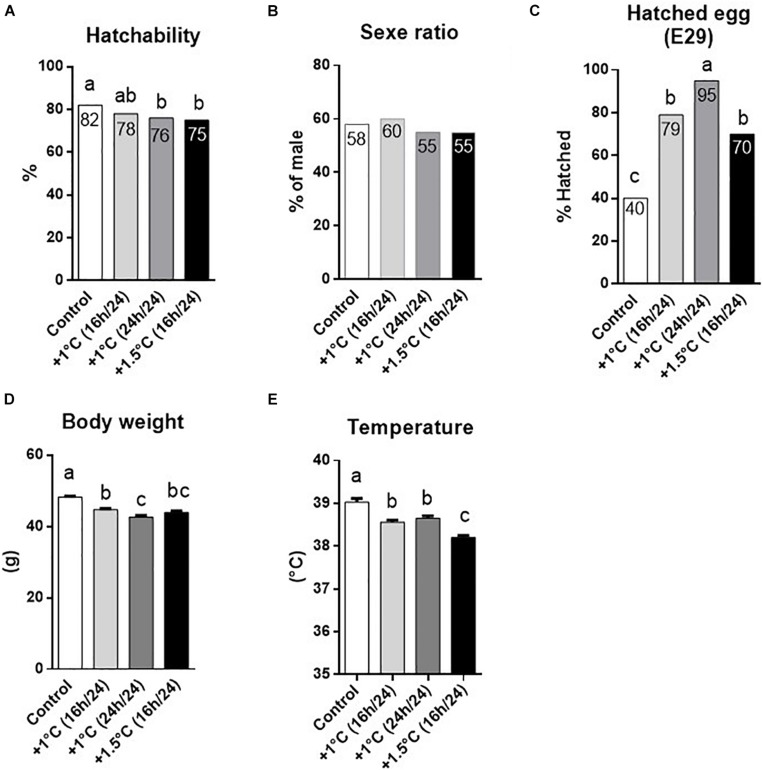
Hatching data. **(A–C)** Percentage of total hatchability **(A)**, sex ratio **(B)**, and E29 hatched eggs **(C)** of mule eggs in control condition, or thermic manipulated conditions. **(D,E)** Mean Body weight **(D)** and body surface temperature **(E)** of male mule duckling 1 day after hatching in control (white bar), +1°C 16 h/24 h (clear gray bar), +1°C 24 h/24 h (dark gray bar) and +1.5°C 16 h/24 h (black bar) groups. Chi^2^ analysis between each group were used for hatchability (*n* = 300–318) **(A)**, sex ratio **(B)**, and percentage of hatched eggs at E29 (*n* = 224–262) **(C)**. Kruskal–Wallis with Dunn’s multiple comparisons test were used for body weight **(D)** and temperature **(E)**, *n* = 124–15, presented with standard error of mean (SEM). Values without common letters were statistically different (*P* < 0.05).

First, the potential impact of incubation temperature on hatching data was examined. Even if no difference was measured between the moderately treated (+1°C 16 h/day) and control groups (*P* > 0.1), the hatching process was clearly affected by the thermal manipulation, as we measured a significant decrease (*P* < 0.05) in hatching success in the thermally manipulated groups with an increase of 1°C 24 h/day and of 1.5°C 16 h/day (76 and 75% of hatchability, respectively vs. 82% for the controls) (Figure 2A). It is interesting to note that these two last TM groups presented exactly the same cumulative increase in temperature (+336°C), while the +1°C 16 h/day group accumulated much less heat during the incubation period (+224°C). No sex-ratio difference was observed between the control and treated groups (Figure 2B), but the mean incubation time was reduced by about one day in all TM groups compared to control (*P* < 0.0001). Indeed, at D29, 70–95% of TM animals hatched against 40% for the control group (Figure 2C).

Finally, significant decreases in total body weight (from 3.48 to 5.56 g) and body surface temperature (between 0.4°C and 0.8°C) were observed at hatching for the three TM groups compared to control (Figures 2D,E). Total body weight no longer differed between control and TM groups at D17 (*P* > 0.1; Table 1), nor did liver, abdominal fat and leg muscle weights (data not shown).

**TABLE 1:** Breeding data.

**A**						
**Average temperature of the body surface (°C)**	**Control**	**+1°C 16 h/24 h**	**+1°C 24 h/24 h**	**+1.5°C 16 h/24 h**	**Test**	***P* value**
D1	39.0 ± 0.1(*a*)	38.6 ± 0.1(*b*)	38.6 ± 0.1(*b*)	38.2 ± 0.0(*c*)	KW	<0.0001
D31	35.2 ± 0.1	35.2 ± 0.1	35.4 ± 0.1	35.4 ± 0.1	KW	0.59
D45	33.8 ± 0.2(*a*)	31.8 ± 0.2(*c*)	32.6 ± 0.2(*b*)	32.3 ± 0.2(*bc*)	ANOVA	<0.0001
D59	33.3 ± 0.2(*a*)	30.5 ± 0.2(*b*)	31.0 ± 0.2(*b*)	30.6 ± 0.2(*b*)	KW	<0.0001
D70	31.9 ± 0.2(*ab*)	32.0 ± 0.2(*ab*)	31.4 ± 0.2(*b*)	32.5 ± 0.1(*a*)	ANOVA	<0.0001
D83	34.8 ± 0.2	35.2 ± 0.2	35.2 ± 0.2	34.5 ± 0.2	KW	0.04
D95	35.0 ± 0.1	35.0 ± 0.1	35.1 ± 0.1	35.2 ± 0.1	ANOVA	0.53

**B**						
**Average body weight (g)**	**Control**	**+1°C 16 h/24 h**	**+1°C 24 h/24 h**	**+1.5°C 16 h/24 h**	**Test**	***P* value**

D1	48.2 ± 0.3(*a*)	44.7 ± 0.3(*b*)	42.6 ± 0.4(*c*)	43.9 ± 0.4(*bc*)	KW	<0.0001
D17	481.5 ± 4.1(*ab*)	479.8 ± 4.4(*b*)	495.2 ± 6.7(*ab*)	506.9 ± 6.7(*a*)	KW	0.026
D31	1669 ± 13(*ab*)	1646 ± 14(*b*)	1713 ± 16(*a*)	1719 ± 17(*a*)	ANOVA	0.0012
D45	2622 ± 22(*b*)	2770 ± 23(*a*)	2833 ± 22(*a*)	2760 ± 26(*a*)	ANOVA	<0.0001
D59	3684 ± 30(*c*)	3808 ± 29(*b*)	3943 ± 29(*a*)	3865 ± 31(*ab*)	ANOVA	<0.0001
D70	4167 ± 36(*b*)	4181 ± 33(*b*)	4351 ± 39(*a*)	4240 ± 35(*ab*)	ANOVA	0.0009
D83	4562 ± 39(*ab*)	4522 ± 38(*b*)	4683 ± 41(*a*)	4576 ± 41(*ab*)	ANOVA	0.03
D95	6102 ± 54	6144 ± 46	6208 ± 51	6191 ± 49	KW	0.37

*(A,B) – Mean body surface temperature (A), and weight (B), of male mule ducks from day 1 post-hatching (D1) to day 95 post-hatching (D95) in control and temperature-manipulated groups. Based on Shapiro test results, Kruskal–Wallis (KW) or one-way ANOVA (followed by Dunn’s or Tuckey’s multiple comparison test comparing the mean of each group on the same day) were performed. Values without common letters were statistically different.*

### Growth Performance and Body Surface Temperature From First Feeding Up to Overfeeding

Changes in body surface temperature and body weight throughout the rearing phase are presented in Table 1. All TM conditions sharply reduced body surface temperature from hatching (between 0.4°C and 0.8°C) until D59 (between 2.3°C and 2.7°C), except at D31, corresponding to the shutdown of heating in the pens. No difference was observed before and after overfeeding.

Thermal manipulation-treated groups compensated for their body weight deficiency in just two weeks post hatching, and even exceeded the controls between D45 and D59 (Table 1). At D70, a significant increase in BW for the group with a rise of 1°C 24 h/day was still observed, but these differences completely disappeared at the beginning of overfeeding period.

These results should be analyzed in conjunction with the daily weight gain, also increased in the TM groups during the D31–D44 period and decreased during the D59–D69 period (Table 2), respectively correlated to a better and worse feed conversion ratio (FCR) compared to the control group (data not shown).

**TABLE 2:** Effect of temperature manipulation on average daily gain (ADG).

**Average daily gain (g/day/animal)**	**Control**	**+1°C 16 h/24 h**	**+1° C 24 h/24 h**	**+1.5°C 16 h/24 h**	**Test**	***P* value**
D1–D17	27.09 ± 0.25(*b*)	27.16 ± 0.27(*b*)	28.31 ± 0.41(*ab*)	28.93 ± 0.40(*a*)	KW	0.0016
D18–D30	91.20 ± 0.72(*ab*)	89.44 ± 0.78(*b*)	93.38 ± 0.79(*a*)	93.20 ± 0.80(*a*)	ANOVA	0.0007
D31–D44	73.26 ± 1.07(*c*)	86.62 ± 1.11(*a*)	86.16 ± 0.97(*a*)	80.68 ± 1.07(*b*)	KW	<0.0001
D45–D58	81.56 ± 1.92(*b*)	80.77 ± 1.26(*b*)	86.95 ± 1.31(*a*)	83.91 ± 1.25(*ab*)	KW	0.0013
D59–D69	48.90 ± 1.53(*a*)	38.77 ± 1.12(*b*)	40.94 ± 1.54(*b*)	38.08 ± 1.39(*b*)	ANOVA	<0.0001
D70–D83	33.54 ± 1.29(*a*)	28.20 ± 1.01(*b*)	26.24 ± 1.16(*b*)	29.85 ± 1.13(*a*)	ANOVA	<0.0001
D84–D95 (OF)	139.3 ± 2.03	142.5 ± 2.64	135.9 ± 2.09	142.4 ± 1.99	ANOVA	0.119

*Daily gain were determined using body weight of animal from day 1 to day 17 (*n* = 122–151), from day 18 to day 30 (*n* = 94–96), from day 31 to day 44 (*n* = 93–96), from day 45 to day 58 (*n* = 92–94), from day 59 to day 69 (*n* = 90–95), from day 70 to 83 (*n* = 88–91) and from day 84 to day 95 (*n* = 46–52). Based on Shapiro test results, Kruskal–Wallis (KW) or one-way ANOVA (followed by Dunn’s or Tuckey’s multiple comparison test comparing the mean of each group on the same period) were performed. Values without common letters were statistically different.*

Finally, all differences in temperature and body weight between treatments had disappeared at the beginning of overfeeding period.

### Plasma Metabolites and Hormones Before and After Overfeeding

The lipid and hormone plasmatic concentrations are presented in Table 3. Before OF (left-hand panel) one TM group (+1°C 16 h/day) presented a significant increase in corticosterone level compared to control, but this difference was no longer observed after OF (right-hand panel).

**TABLE 3:** Plasma data.

**Average plasma concentrations**	**Pre-overfeeding (D84)**				**Post-overfeeding (D95)**				**OF effect (*p* value)**	**TM effect (*p* value)**	**Interaction (*p* value)**
					
	**Control**	**+1°C 16 h/24 h**	**+1°C 24 h/24 h**	**+1.5°C 16 h/24h**	**Control**	**+1°C 16 h/24 h**	**+1°C 24 h/24 h**	**+1.5°C 16 h/24 h**			
Glucose (g/L)	2.22 ± 0.08	2.41 ± 0.09	2.41 ± 0.07	2.34 ± 0.09	3.00 ± 0.17	3.11 ± 0.11	2.92 ± 0.08	3.00 ± 0.13	<0.0001	ns	ns
Triglycerides (g/L)	0.94 ± 0.08	1.17 ± 0.09	1.14 ± 0.11	1.02 ± 0.05	3.74 ± 0.28	4.26 ± 0.24	3.60 ± 0.21	3.79 ± 0.22	<0.0001	ns	ns
Cholesterol (g/L)	1.80 ± 0.14	2.02 ± 0.15	2.06 ± 0.11	1.85 ± 0.09	3.18 ± 0.13	3.54 ± 0.15	3.66 ± 0.15	3.31 ± 0.17	<0.0001	ns	ns
FFA (g/L)	0.26 ± 0.03	0.27 ± 0.02	0.23 ± 0.02	0.29 ± 0.02	0.23 ± 0.01	0.24 ± 0.02	0.22 ± 0.01	0.22 ± 0.02	0.0165	ns	ns
Corticosterone (ng/mL)	81 ± 9	130 ± 10	102 ± 10	99 ± 12	42 ± 6	51 ± 5	45 ± 7	41 ± 5	<0.0001	0.0064	ns
Free T3 (pmol/L)	3.79 ± 0.17	3.71 ± 0.17	3.69 ± 0.21	4.42 ± 0.19	6.82 ± 0.28	7.13 ± 0.29	6.09 ± 0.25	6.89 ± 0.31	<0.0001	0.0147	ns
Free T4 (pmol/L)	9.36 ± 0.26	9.50 ± 0.43	10.01 ± 0.21	9.89 ± 0.40	6.75 ± 0.64	7.70 ± 0.79	8.39 ± 0.58	9.09 ± 0.55	<0.0001	0.0365	ns
Ratio T3/T4	0.40 ± 0.01	0.41 ± 0.03	0.41 ± 0.04	0.41 ± 0.02	1.00 ± 0.10	0.88 ± 0.10	0.75 ± 0.09	0.81 ± 0.06	<0.0001	ns	ns

*Mean plasma concentrations of different metabolites in male mule ducks before (Pre-OF) and after overfeeding (post-OF). Two-way ANOVA (followed by Tuckey’s multiple comparison test) was performed. Values without common letters were statistically different. ns, non-significant.*

As expected, OF strongly altered the lipid parameters. Indeed, glucose, triglycerides and cholesterol concentrations rose sharply after 21 forced meals in all groups, while free fatty acids decreased slightly. OF also significantly affected plasma hormone levels, as a substantial increase in T3 and a large decrease in corticosterone were measured, but no differences were observed between the TM and control groups. T4 plasmatic concentrations also dropped slightly after OF compared to before OF, but no difference was observed between the TM and control groups.

### Body Composition Before and After Overfeeding

**FIGURE 3:**
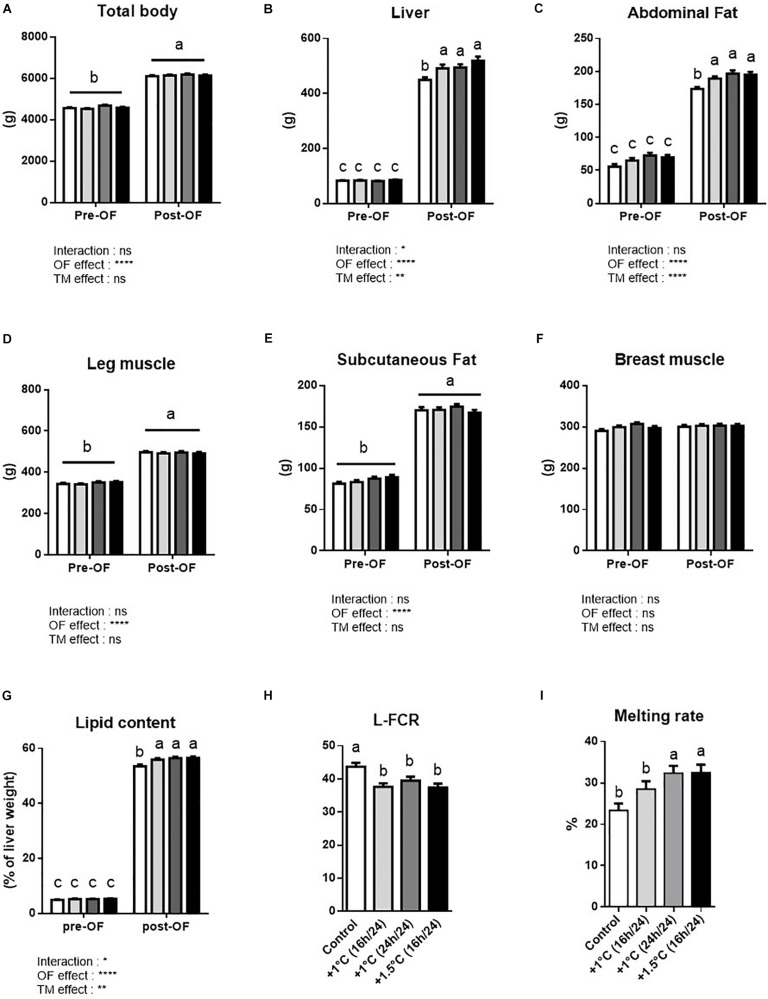
Pre and post over-feeding data. **(A–F)** Average weight of body **(A)**, liver **(B)**, abdominal fat **(C)**, leg muscle **(D)**, subcutaneous fat **(E)**, and breast muscle **(F)** of male mule ducks at D84 pre-OF and D95 post-OF in control (white bar), +1°C 16 h/24 h (clear gray bar), +1°C 24 h/24 h (dark gray bar) and +1.5°C 16 h/24 h (black bar) groups. **(G)** Estimated lipid content in liver of pre-OF (*n* = 37–39) and post-OF (*n* = 47–52) in control (white bar), +1°C 16 h/24 h (clear gray bar), +1°C 24 h/24 h (dark gray bar) and +1.5°C 16 h/24 h (black bar) groups, established as described in section “Materials and Methods.” Two-way ANOVA and Tukey’s multiple comparisons test were performed **(A–G)**. *n* = 90–95 for pre-OF and *n* = 48–52 for post-OF **(A)**, *n* = 36–38 for pre-OF and *n* = 47–52 for post-OF **(B–G)**. **(H)** Liver-Food conversion ratio (L FCR) per bird was determined using the individual daily consumption and the liver weight gain during OF (measured using each group pre-OF average liver weight and post-OF individual liver weight). One-way ANOVA and Tuckey’s multiple comparisons test were performed (*n* = 46–52). **(I)** *Foie gras* melting rate in overfed male mule ducks in control, (white bar), +1°C 16 h/24 h (clear gray bar), +1°C 24 h/24 h (dark gray bar) and +1.5°C 16 h/24 h (black bar) groups (*n* = 47–52). Kruskal–Wallis and Dunn’s multiple comparisons test were performed (*n* = 47–52). All data are presented with SEM. ^∗^*p* < 0.05; ^∗∗^*p* < 0.01; ^∗∗∗∗^*p* < 0.0001. Values without common letters were statistically different (*P* < 0.05), ns, non-significant.

The impacts of overfeeding and TM treatments are shown in Figure 3. First, all groups exhibited a higher total body weight, and liver, abdominal fat, leg muscle and subcutaneous fat weights after OF compared to before OF (Figures 3A–E). Interestingly, the effect of TM was only highlighted for liver and abdominal fat weights, which were significantly enhanced in all TM-treated groups after OF compared to their own control (Figures 3B,C). This increase in liver weight was associated with a greater increase in lipid content in the TM groups after OF (Figure 3G). However, despite the TM groups having better L-FCR than the controls (Figure 3H), we measured an increase in the melting rate for the TM-treated groups + 1°C 24 h/day and + 1.5°C 16 h/day (Figure 3I), corresponding to the cumulative temperature rise of + 336°C.

**FIGURE 4:**
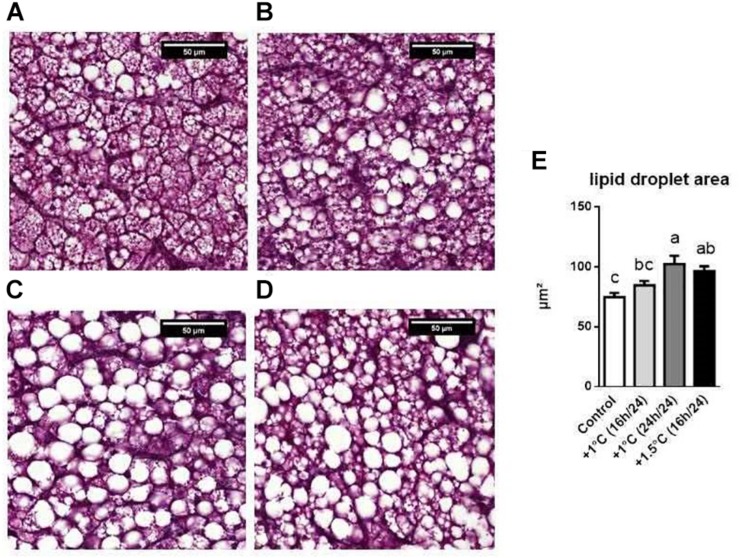
Histological analysis. **(A–D)** Representative images of lipid droplet in the liver of the different groups: control **(A)**, +1°C 16 h/24 h **(B)**, +1°C 24 h/24 h **(C)** and +1.5°C 16 h/24 h **(D)**. Scale bars: 50 μm. **(E)** Mean of lipid droplet area above 48 μm^2^ of male mule ducks post-OF (*n* = 14, the analysis was done on two different images of each sample for a total of seven samples per group). Data are presented ± SEM. One-way ANOVA (followed by Tuckey’s multiple comparison test) was performed. Values without common letters were statistically different (*P* < 0.05).

Afterward, although we did not measure any change in the grade of liver steatosis assessed by Masson’s trichrome coloration (data not shown), we recorded a substantial rise in the size of vesicular lipid droplets in the same two treated groups, which reflects an increase in the melting rate after OF compared to the control group (Figure 4).

## Discussion

Several studies have previously shown that embryonic thermal manipulation (TM) could enhance adult poultry performance characteristics, such as thermoregulation during acute heat stress and breast muscle yield (Piestun et al., 2011, 2013; Al-Rukibat et al., 2017). For the first time, we have shown that a moderate increase in incubation temperature during the second half of embryogenesis could improve foie gras production (by up to 15%) in adult male mule ducks.

### Early Impact of Embryonic TM

The actual impact of embryonic TM on hatchability remains to be clarified, as the results can be totally inconsistent according to the temperature variation, period and duration of the treatment (Collin et al., 2005; Piestun et al., 2008a; Loyau et al., 2013; Narinç et al., 2016; Al-Rukibat et al., 2017). Furthermore, most of the previous studies were conducted on chicken, raising the possibility that different species could respond differently to TM. Firstly, our results confirm previous data showing early hatching in the event of increased incubation temperature for all treated groups (DuRant et al., 2010, 2011, 2013; Hepp and Kennamer, 2012). Secondly, higher discontinuous (+ 1.5°C 16 h/day) and lower but constant (+ 1°C 24 h/day) increases in incubation temperature significantly reduced hatching success compared to the control group. On the contrary, a moderate but discontinuous temperature rise (+ 1°C 16 h/day) did not affect hatchability. These results suggest that in mule ducks as in broilers (Piestun et al., 2008a), continuous TM has a more negative effect on hatching performance than intermittent TM, and reveal that ducks are more sensitive to moderate temperature increases during embryogenesis than broilers. However, several studies reported that hatching success in mule ducks was generally measured at between 70 and 80% of fertile eggs (Brun et al., 2005; Marie-Etancelin et al., 2008), which implies that even though this capacity is clearly affected by heat treatment, hatching performance remains in the same range (at between 75 and 82%). Concerning the sex ratio, TM did not change the imbalance induced by mule hybrid generation, leading to an increase in female embryonic mortality (Batellier et al., 2004).

As frequently described for chickens and turkeys, the body weight (Piestun et al., 2008a; Al-Rukibat et al., 2017) and body surface temperature (Loyau et al., 2013; Al-Zghoul et al., 2015) of TM ducks were significantly lower than those of the controls on hatching day. However, while lower body surface temperatures were maintained in all treated groups until 59 days of breeding, TM ducks compensated for their growth retardation in as few as 17 days after birth, and even exceeded the weight of control ducks between 45 and 59 days of age. Finally, these disparities diminished at the end of breeding to reach control level, with differences no longer observed at the start of the overfeeding procedure.

Thyroid hormones participate in the regulation of heat production and thermo-tolerance acquisition in poultry, and embryonic TM obviously affects their plasmatic concentrations in chickens, both during incubation and after hatching (Piestun et al., 2009). However, the mid-term consequences of embryonic TM on thyroid activity are still controversial. Indeed, plasma measurements of T3 and T4 concentrations in chicken raised under normal temperature conditions but previously subjected to embryonic TM may give rise to conflicting results. Piestun et al. (2011) showed a decrease in T3 and T4 plasma level a few weeks after hatching, Loyau et al. (2013) measured a drop in the T3/T4 concentration ratio in 28-day-old chickens but no longer afterward, while another study highlighted an increase in both hormones until 42 days post-hatching (Al-Zhgoul et al., 2013). In the present study, embryonic TM did not alter T3 and T4 plasma concentrations in ducklings at two weeks of age (data not shown). These results are consistent with previous research (Piestun et al., 2008b) and suggest that thyroid axis activity is not strongly altered in the absence of additional thermal challenge, even if body surface temperatures were reduced for several weeks.

Very few studies of the impacts of TM on ducks have been carried out so far. Nevertheless, Liu et al. (2015) have demonstrated that a continuous rise of 1°C in the incubation temperature during the second half of embryogenesis led to an increase in body weight and liver relative weight at 2 weeks post-hatching. These results prompted us in the present study to examine the hypothesis of enhanced hepatic metabolism after TM in mule ducks, which could be particularly beneficial for foie gras production. However, these results were not reproduced in mule ducks at two weeks of age, as the total body and liver weights remained unchanged for all treated groups (data not shown).

### Effect of TM Manipulations on Plasma Parameters Before and After Overfeeding

The consequences of embryonic TM in adult poultry have often been studied in the restricted context of acute heat stress response capacity. The aim of this trial was to evaluate liver fattening in overfed adult mule ducks previously treated with three types of embryonic TM. The four groups were submitted to the same overfeeding (OF) conditions for 11 days at 12 weeks of age (21 meals). Since thermally manipulated Pekin ducks present an increase in plasma cholesterol two weeks after hatching (Wang et al., 2014), and given that the differential genetic ability of OF-induced fattening of the liver has been related to distinct levels of triglyceridemia or circulating free fatty acids (Hermier et al., 2003; Tavernier et al., 2017), we evaluated the impact of embryonic TM on plasma metabolite concentrations in mule ducks. We then found that even if OF strongly increases the plasma content of glucose, TG, and cholesterol as previously shown (Davail et al., 2003; Hermier et al., 2003; Chartrin et al., 2006; Marie-Etancelin et al., 2011), no effect of incubation temperature on these variables was observed, around hatching (data not shown) and before or after OF. These data again suggest that incubation temperature has no impact on the metabolic parameters of plasma in the absence of further thermal challenge.

Overfeeding also induced a sharp decrease in the corticosterone content of plasma in mule ducks in all groups compared to animals collected prior to overfeeding. As corticosterone may reflect the stress response (Harvey et al., 1980), these results are consistent with those of a previous study on overfed mule ducks, revealing habituation to physical restraint during OF (Guémené et al., 2006). Again, no effect of TM was measured at this stage compared to the overfed control group.

In the same way, thyroid hormones were also altered by OF, since we recorded a significant increase in triiodothyronine (T3) and a slight decrease in thyroxin (T4) content in the plasma of all overfed groups compared to animals sampled before OF. Since the plasma content of T3 (the active form) may result from both thyroid secretion and peripheral deiiodination of T4 (Pittman et al., 1971; Surks et al., 1973), in our trial, it is not surprising to observe a decrease in T4 at the same time as an increase in T3. In both humans and rodents, it has long been known that T3 production is stimulated by overfeeding (Danforth et al., 1979; Katzeff, 1990), which is supposedly responsible for an increase in energy expenditure by non-shivering thermogenesis in order to limit weight gain (Acheson et al., 1984; Stock, 1989). A decrease in diet-induced thermogenesis has also been associated with obesity (Trayhurn, 2017). Therefore, in the context of foie gras production in ducks, and based on several data showing better thermoregulation in thermally manipulated birds (Al-Rukibat et al., 2017; Zaboli et al., 2017), we hypothesized that embryonic TM could reduce OF-induced thermogenesis due to downregulation of the thyroid axis and thus improve liver fattening. However, in the present study, no programing effect was revealed on the T3 and T4 concentrations compared to the control group or on the T3/T4 ratio, despite some slight variations between TM subgroups. In addition, no difference in body surface temperature was observed after OF.

However, even though we did not confirm the role of T3 hormone and thermogenesis in the improvement of liver fattening observed in thermally manipulated ducks, it might be interesting to measure the OF response in summer, when the thermoregulatory capacity is challenged.

### TM Manipulations Significantly Affect Overfeeding-Induced Liver Fattening

Finally, as expected, overfeeding induced a strong increase in total body, adipose tissue, liver and leg muscle weights in all groups (Davail et al., 2003; Hermier et al., 2003; Lo et al., 2017). Interestingly, all TM conditions specifically enhanced liver and abdominal fat weights after OF compared to their own controls. In liver, this rise occurred in parallel with an increase in lipid content in the three treated groups compared to control, suggesting that lipid metabolism remains affected for several months after the change in temperature. These data strongly suggest that in mule ducks, an increase in incubation temperature during embryogenesis results in true metabolic programing. The liver fattening was, however, combined with a higher melting rate in two of the three treated groups, implying that the primary conditions of embryonic TM could be optimized to obtain the best-quality foie gras, in particular by choosing a moderate discontinuous thermal manipulation. In addition, histological analysis also led us to conclude that embryonic TM may modify the type of hepatic fattening during OF, since we measured a significant increase in large vesicles in the same groups that presented a high melting rate, confirming previous results (Theron et al., 2011). In the future, it could be interesting to examine this observation in detail in order to determine the existence of a potential correlation between both characteristics, which could imply differential metabolic mechanisms such as cell hyperplasia or hypertrophy.

It is particularly interesting to note that the two most challenging TM conditions (+ 1°C 24 h/day and + 1.5°C 16 h/day) – fortuitously corresponding to the same cumulative increase in temperature (a total of + 336°C) – led to the same phenotype in term of hatchability, the surface area of lipid droplets and liver melting rate.

These results suggest that foie gras production could be optimized extremely precisely by embryonic thermal manipulation, and that the cumulative rise in temperature should probably not exceed 336°C. Accurate knowledge of lipid and carbohydrate metabolism during embryogenesis may also be useful in choosing the best programing period during which the duration and the level of temperature change could be modulated without exceeding this threshold. Indeed, this trial has shown the strict positive impact of a cumulative temperature increase of 224°C, corresponding to the + 1°C 16 h/day group, which enabled the same increase in foie gras production as the other TM groups without any negative impact on hatchability or the melting rate. These results open up many opportunities for improving foie gras production.

## Conclusion

In this way, a moderate embryonic thermal manipulation induced accelerated development, a potential reduction in thermogenesis suggested by reduced body surface temperature, and an increase in hepatic steatosis after OF. We have also confirmed a potential link between hepatic lipid vesicle size and the melting rate, which is one of the most important traits of the technological performance of foie gras. To our knowledge, this is the first study to demonstrate a positive impact of embryonic thermal manipulation on foie gras production in overfed mule ducks. As a precursor, this trial not only opens a broad new field of agronomic research but also arouses fundamental interest in the underlying mechanisms involved in “thermal programing.”

## Data Availability Statement

All data used in this study are available from the corresponding authors upon reasonable request.

## Ethics Statement

The animal study was reviewed and approved by the Care and Use of Animals of the Grand Sud-Ouest (No. 73).

## Author Contributions

MH, SP, AC, SD, MM, and WM conceived and designed the study. WM conducted all the experiments and analyses with the help of MH, TP, KR, KG, and AT. M-DB supervised the whole breeding, overfeeding, and slaughtering phases. All authors reviewed the manuscript.

## Conflict of Interest

The authors declare that the research was conducted in the absence of any commercial or financial relationships that could be construed as a potential conflict of interest.

## Footnotes

1 www.graphpad.com
